# Legacy Pollutants are Declining in Great Skuas (*Stercorarius skua*) but Remain Higher in Faroe Islands than in Scotland

**DOI:** 10.1007/s00128-016-1856-x

**Published:** 2016-06-21

**Authors:** Sjurdur Hammer, Ruedi G. Nager, Sarah Alonso, Rona A. R. McGill, Robert W. Furness, Maria Dam

**Affiliations:** Institute of Biodiversity, Animal Health & Comparative Medicine, University of Glasgow, Glasgow, UK; NERC Life Sciences Mass Spectrometry Facility, Scottish Universities Environmental Research Centre, Rankine Avenue, East Kilbride, G75 0QF Scotland, UK; MacArthur Green, 95 South Woodside Road, Glasgow, UK; Environment Agency, Traðagøta 38, Argir, FO-165 Faroe Islands

**Keywords:** Persistent organic pollutants, Bioaccumulation, Faroe Islands, Eggshell thickness, Eggshell, Stable isotope analysis

## Abstract

To monitor environmental pollutants in Faroese biota, samples from a top predator were analysed and put into a spatial and temporal context. Analysis of 20 Great Skua eggs sampled in 2012 from the Faroe Islands showed >70 % lower concentrations of legacy persistent organic pollutants (POPs) than in samples analysed in 1977. The 2012 Faroese eggs showed higher concentrations than for eggs in Shetland from about the same period (2008). Eggshells were analysed for sub-lethal effects but there were no detectable effects of legacy POP levels on eggshell colour or thickness. A temporal decline in legacy POPs would indicate a reduction in the general pollutant levels present in the environment as has been shown in other areas of the North Atlantic, but there are significant geographic differences in POPs levels likely due to differences in diet resulting in significantly different exposures on a relatively limited spatial scale.

Top predators are particularly exposed to environmental pollutants which magnify in the food-chain (Newman 2010), and as such are often used as monitors of pollution levels in the environment (Furness et al. 1997). Due to the high trophic position of seabirds, they have been found to accumulate lipid-soluble persistent organic pollutants (POPs). There may, however, be strong geographic differences in pollutant concentrations found in top predators due to various factors such as proximity to pollutant sources, latitudinal variation in atmospheric deposition, and spatial differences in diet (Bourgeon et al. 2013).

Eggs from Great Skuas (*Stercorarius skua*) a top predator in the Faroese marine ecosystem were sampled in 1977 showed exceptionally high concentrations in total PCB, DDT and DDE and four and five orders of magnitude higher concentrations of PCB and DDE respectively, compared to eggs of Northern Fulmars (*Fulmarus glacialis*) (Bloch et al. 1987). With the implementation of regulations restricting the use of PCBs in the 1970s and the ban under the Stockholm Convention came into action in 2004, several POPs have been phased out of use (UNEP 2009). However, they may still persist in the environment, despite production having been halted or reduced, and are referred to as *legacy POPs*. Moreover, large volumes of PCBs applied prior to the restrictions are today part of buildings and the technosphere in general and leak into the environment (Breivik et al. 2002). Considering the historic high levels in Faroese Great Skua eggs, an evaluation of the pollutant status of Great Skuas in the Faroe Islands (Faroes hereafter) is timely and important. A recent study from Shetland comparing Great Skua eggs from 1980 and 2008 showed a significant decline in most legacy POPs (Leat et al. 2011). The decline was greatest for the least persistent pollutants such as DDT and the more readily metabolized PCB congeners (Group IV in Borgå et al. (2005), suggesting a reduced presence in the wider marine ecosystem of some of the legacy POPs (Leat et al. 2011). The aim of the present analysis of eggs collected in the Faroes in 2012 is to determine if there is also a significant decline in legacy POPs in the Faroese environment, similar to Shetland.

Some pollutants, such as DDT and DDE influence calcium metabolism of birds, and have been shown to cause eggshell thinning (Ratcliffe 1970). A thinning of eggshells is widely associated with reduced hatching success (Beyer et al. 1996). The concentrations in Faroese Great Skua eggs in the 1970s was at a level that caused eggshell thinning in other seabird species such as Ivory Gull (*Pagophila eburnea*) (Miljeteig et al. 2012). The potential of DDT and DDE to interfere with the organisms’ calcium metabolism has also been proposed to have a measurable effect on eggshell colour (Jagannath et al. 2008), and a recent study on Herring Gulls (*Larus* argentatus) from the Great Lakes detected a relationship between eggshell colour and POPs levels in the eggs (Hanley and Doucet 2012). Therefore, as a part of our analysis of pollutant concentrations in Great Skua eggs, we also measured eggshell thickness and colour and tested if there was a relationship with the egg’s pollutant load.

There was a significant geographic difference in POPs levels between Great Skua eggs from Shetland and Faroes in the 1970s (Bloch et al. 1987; Leat et al. 2011). Here we will also investigate the geographical variation in contemporaneous POP levels between Faroes and Shetland using published data for Shetland from Leat et al. (2011) and freshly collected eggs from the Faroes. Among the factors that could contribute to the geographic differences in POPs concentrations is difference in diet between breeding areas. We expect populations that forage on a higher trophic level (measured as δ^15^N stable isotope ratio of the egg material) to accumulate higher POP levels due to bio-accumulation of contaminants through the food web.

## Materials and Methods

In 2012, 20 eggs from 20 different nests were collected from the Great Skua colony at Skúvoy, Faroe Islands (61°46′N 6°49′W). Nesting territories were located and selected randomly throughout the colony, by observing presence and aggressive behaviour of at least one parent. Within approximately 1 week after laying, single eggs were collected from two-egg clutches only, and were refrigerated overnight. The next day, eggs were opened along the long axis and the egg content emptied, homogenized and frozen at −20°C until analysis. Eggshells were dried at air temperature and then stored in a dry and dark condition.

The POPs analysis on egg content was performed by the Centre de Toxicologie du Québec (Québec, Canada) (method ref: E-448) which is accredited under ISO17025 and participates in the Arctic Monitoring Assessment Programme. The samples were enriched with internal standards (hexachlorobenzene-^13^C_6,_ α-HCH-^13^C_6_, oxychlordane-^13^C_10_, trans-nonachlor-^13^C_10_, p,p′-DDE-^13^C_12_, PCB 141-^13^C_12_, PCB 153-^13^C_12_, PCB 180-^13^C_12_, parlar 26-^13^C_10_ and parlar 50-^13^C_10_), mixed with dichloromethane and chemically dried using sodium sulphate. The compounds were then extracted in an ultrasonic bath followed by a filtration. A subset of the sample was used to determine lipid percentage by gravimetry. The sample for analyses was concentrated by evaporation and purified using gel permeation chromatography (GPC) and cleaned-up on Florisil column. The extracts were analysed using an Agilent 5973 gas chromatograph equipped with a PTV injector and an AutoSpec Ultima High Resolution Mass Spectrometer (HRMS) from Waters/Micromass. The HRMS was operated at Resolution 10,000 at 10 %. All chromatography was performed on a 60 m, 0.25 mm DB5 MS capillary column. For quality assurance, reference material (fish tissue SRM-1947) was used, 15 % of samples were run as dublicates, and corn oil was used for method blanks. The analysis was done for the following compounds: PCB congeners 28, 52, 99, 101, 105, 118, 128, 138, 153, 156, 163, 170, 180, 183, 187, PCB Aroclor 1260, o,p′-DDE, α-chlordane, γ-chlordane, cis-nonachlor, trans-nonachlor, hexachlorobenzene, Mirex, oxychlordane, p,p′-DDE, o,p′-DDT, p,p′-DDT, β-HCH, Toxaphene parlar 26, 32, 50, 62. The results were reported as µg/kg lipid of tissue analysed, along with the tissue lipid content in wet weight (ww) percent. In order to control for variation in lipid content during data analysis each sample was multiplied by lipid percentage and is reported as µg/kg ww tissue. The limit of detection (LOD) was determined as signal to noise ratio of 3. A sample with a concentration of 4 to 10 times the estimated LOD was analysed with 10 replicates. The calculated LOD was the value equivalent to thrice the standard deviation of the 10 replicates. The LOD was between 0.1 to 10 µg/kg lw for all samples. The LOD was adjusted to weight and lipid content of each sample, providing different LODs for each sample. 100 % Gamma chlordane and 90 % of PCB congener 52 samples were below their detection limits of 0.004 µg/kg ww and 13.6 µg/kg ww, respectively,and these compounds were excluded from further analysis. The sum of PCB congeners is referred to as sum14PCB.

To assess trophic level of Faroese Great Skuas during egg formation, stable isotope analysis of the egg content was undertaken at the NERC Life Sciences Mass Spectrometry Facility in East Kilbride, UK. The egg material was oven-dried at 40°C for >24 h. Stable nitrogen isotope analyses can be used as a proxy for the trophic level of ingested prey (Hobson and Welch 1992), and were performed by continuous flow isotope ratio mass spectrometry (CF-IRMS) on an average of 0.8 ± 0.1 mg of sub-sampled material loaded into tin cups and combusted in a Costech ECS 4010 elemental analyser coupled to a Thermo Finnigan Delta Plus XP mass spectrometer. Lab standards (gelatin, alanine and glycine) were run between every 10 samples to correct for instrument drift. Isotope ratios are expressed as parts per thousand (‰) according to the equation *δX* = [(R_sample_/R_standard_)-1] × 1000, where *X* is ^15^N, R is the ^15^N/^14^N, and R_standard_ is the ratio of the international references AIR for nitrogen. Lab standards were calibrated with international reference standards and the measurement precision is calculated as the standard deviation of multiple measurements of internal lab standard material (tryptophan) which was ±0.209  ‰.

Eggshell thickness, included the inner and outer membrane, was measured using a micrometer with a ballpoint tip, to the nearest 0.01 mm at three different points along the long axis of the egg (pointed, equator and blunt). Measurements at each location along the long axis were repeated three times and the repeatability of the measurements at each location of the egg were calculated using Intraclass Correlation Coefficient (ICC package, Wolak et al. 2012; in R version 3.1.2, R Core Team 2014). Measurement repeatabilities were high at each of the locations – ICCs were between 0.90 and 0.92. In order to compare with previously reported thickness values (measured without membranes) we also measured eggshell thickness without membranes where possible on a subset of samples.

The eggshell background colour was determined using photographs in RAW format taken by a digital SLR camera (Nikon D7000). The photographs were taken on the intact egg outside in ambient light during the middle of the day, with the eggs placed on a flat platform with colour reference material (X-rite ColorChecker Passport, Michigan, US) on each photograph. Care was taken to shelter each photograph from direct sunlight. From the image we recorded red (R), green (G) and blue (B) values using the Pixel Inspector Tool plugin in ImageJ (Rasband 2014) from six different pixels of the standardised grey colours on the ColorCheckerPassport and from six different pixels at the blunt end of the egg avoiding maculation and dirt. To control for the effect of variation in light condition on colour (linearization) we used the standardised greyscale colour from the colour reference material where RGB values are known. Known and observed RGB values of the standardised grey colours were regressed against each other and the resulting correction equation applied to the RGB values of the eggshell. It has been suggested that colour data are better interpreted as ratios as opposed to absolute values (Bergman and Beehner 2008), so we analysed R:G as a ratio.

Comparison with published data was done by two-sample *t* tests where appropriate, and Mann–Whitney U tests where the data did not fulfill the normality criteria. Bonferroni correction was applied where multiple *t* tests were carried out on the different POP compounds from the same eggs. For testing differences in thickness between locations on the eggshell, a linear mixed effect model was created using lme4 package in R (Bates et al. 2015). A paired *t* test was used to compare eggshell thickness measurements with and without membranes. To test for associations between POPs concentration and eggshell thickness and color the Spearman ranked correlations were used. We tested correlations between POP compounds and trophic level (δ^15^N). All statistical tests were done in R version 3.1.2 (R Core Team 2014). Means ±1 standard deviation are reported and two-tailed *p* < 0.05 was taken as statistically significant.

## Results and Discussion

The Faroese eggs collected in 2012 had lower DDT, DDE and sum of PCB levels than eggs in 1977 (Table 1). The concentrations of other compounds in Faroese eggs from 2012, (not measured in 1977) such as ß-HCH, HCB, Mirex,Cis-Nonachlor, Oxychlordane, Toxaphene parlar 26, 50, 62, were also significantly higher than in contemporary eggs from Shetland and none of the compounds were significantly lower in Faroes compared with Shetland (Table 1). The reduction in concentrations of legacy POPs from 1977 to 2012 (*p* < 0.01, Table 1) is so large that we can be confident that this reflects a real and considerable decline and is not attributed to differences in analytic approach. A similar temporal decline in legacy POPs concentrations has also been seen in other marine animals such as black guillemot (*Cepphus grylle*) eggs, juvenile pilot whale (*Globicephala melas*) blubber and cod (*Gadus morhua*) from the Faroes (Nielsen et al. 2014). A decline in legacy POPs in the marine environment over the last few decades has also been found in other regions in the North Atlantic and Arctic (Helgason et al. 2008; Muir and de Wit 2010). The relatively greater decline of DDT (99.5 %) in relation to DDE (70.2 %, Table 1) could be explained by the restriction of DDT use as pesticide, while its metabolite—DDE—persists in the environment for longer than the parent compound (Peakall 2007). PCBs also declined by 63.6 %. However caution is needed when comparing PCB data between 1977 and 2012 because different quantification methods were used. The egg samples collected by Bloch et al. (1987) were not available to re-analyse alongside contemporary samples. Nevertheless, we assume the sum PCB as quantified with Clophen A60 in 1977 is broadly comparable to single congener quantification Aroclor 1260. The sum14PCB congeners analysed in the 2012 samples is reported in Table 1, however for comparison with Leat et al. (2011) which did not include congener 163 in its sum14PCB this congener was excluded.

**Table 1:** Mean (standard deviation) and median (range) concentrations (µg/kg wet weight) of organochlorines assayed in Great Skua eggs from the Faroes in 1977 (Bloch et al. 1987), from the Faroes in 2012 (this study) and from Shetland 2008 (Leat et al. 2011)

	Faroe Islands								Shetland	
	1977 (n = 19)		*t* value	*p* value	2012 (n = 20)		*t* value	*p* value	2008 (n = 29)	
	Mean (SD)	Median (min–max)			Mean (SD)	Median (min–max)			Mean (SD)	Median (min–max)
Sum14PCB					5419 (3604)	4530 (1205–12,307)	**4.58**	<0.001	2405 (1829)	1614 (620–7492)
PCB, Aroclor 1260	35,800 (17,614)	32,000 (13,300–96,500)	**12.68**	<0.001	13,100 (9100)	10,660 (2592–31,950)				
p,p′-DDE	6121 (2829)	5500 (1900–16,000)	**7.24**	<0.001	1823 (1212)	1543 (394–4691)	**3.62**	<0.001	924 (908)	598 (254–4588)
p,p′-DDT	7226 (3371)	7000 (2300–18,700)	**31.92**	<0.001	37.8 (24.9)	30.2 (7.2–120.7)	**4.06**	<0.001	18 (15)	15.8 (2.5-63)
ß-HCH					5.5 (5.1)	4.5 (1.2–20.6)	**w** **=** **477**	<0.001	1.8 (1.5)	1.4 (ND–8.6)
HCB					84 (56)	64 (29–223)	**5.11**	<0.001	23 (18)	20 (ND–88)
Mirex					160 (119)	134 (34–459)	**8.46**	<0.001	24 (20)	20 (3.3–110)
Cis-nonachlor					31.6 (18.8)	23.4 (10.8–81.9)	2.09	0.043	22 (11)	20 (7.6–53)
Trans-nonachlor					71 (37)	58 (29–149)	1.14	0.258	103 (83)	77 (6.5–358)
Oxychlordane					176 (148)	126 (34–564)	**7.00**	<0.001	28 (42)	15 (ND–207)
Toxaphene, Parlar no. 26					61 (35)	52 (26–170)	**4.90**	<0.001	32 (15)^b^	26 (17–56)^b^
Toxaphene, Parlar no. 50					144 (76)	116 (65–355)	**5.59**	<0.001	72 (31)^b^	67 (39–125)^b^
Toxaphene, Parlar no. 62					33 (23)^a^	27 (11–105)^a^	**2.76**	<0.001	20 (13)^b^	16 (5.4–47)^b^
Toxaphene, Parlar no. 32					1.7 (0.7)	1.8 (0.8–3.2)				
Alpha-chlordane					4.9 (2.4)	4.8 (1.1–9.2)				
Gamma-chlordane					ND	ND				
PCB, IUPAC # 28					5.5 (3.3)	4.8 (1.5–14.9)				
PCB, IUPAC # 52					ND	ND (ND–12.07)				
PCB, IUPAC # 99					123 (90)	96 (23–321)				
PCB, IUPAC # 101					42 (37)	31 (12–156)				
PCB, IUPAC # 105					63 (42)	48 (14–156)				
PCB, IUPAC # 118					306 (208)	255 (59–710)				
PCB, IUPAC # 128					82 (54)	61 (19–189)				
PCB, IUPAC # 138					656 (422)	536 (135–1420)				
PCB, IUPAC # 153					1868 (1330)	1504 (362–4686)				
PCB, IUPAC # 156					80 (54)	70 (18–185)				
PCB, IUPAC # 163					102 (84)	84 (23–336)				
PCB, IUPAC # 170					354 (261)	267 (81–910)				
PCB, IUPAC # 180					1214 (833)	940 (319–2911)				
PCB, IUPAC # 183					217 (142)	180 (50–497)				
PCB, IUPAC # 187					305 (234)	269 (77–1050)				

For the *t* test and Welch test the data were log transformed. Comparisons between 1977 and 2012 of Faroes eggs and between Faroese eggs in 2012 and Shetland eggs in 2008 are shown with significant differences after Bonferroni correction (corrected *p* = 0.0033) highlighted in bold

^a^n = 18, ^b^ n = 10

The linear mixed effect model showed the mean eggshell thickness to vary along the longitudinal axis (t = 4.30). A Tukey posthoc test showed the pointed end 0.32 mm (±0.028) was signficantly thicker than the blunt end 0.31 mm (±0.023) (*p* < 0.001), but the difference was not significant between pointed end and the equator 0.31 mm (±0.026) (*p* = 0.58). Other studies have shown similar variation in eggshell thickness along the length axis with thinner shells at the blunt end than at the equator and pointed end (e.g. Maurer et al. 2012). Since the difference was small between the locations we used the mean of the eggshell thickness across all three positions for further analysis and the mean eggshell thickness of Faroese Great Skua eggs in 2012 was 0.32 mm (±0.022). Bloch et al. (1987) reported eggshell thickness of 0.28 mm (±0.016) for 19 Great Skua eggs sampled in the Faroes in 1977, although location of thickness measure was not specified. However these measurements were taken without membranes. In our sample we found a difference between shell thickness with and without the membranes of on average 0.06 mm (±0.028, n = 17 eggs; paired- t = 11.84, *df* = 16, *p* < 0.001), which means that the membranes included in the shell thickness measurement could potentially account for the apparent difference in eggshell thickness between 1977 and 2012. There was no significant correlation between DDE (Spearman r_s_ = −0.13, n = 20, *p* = 0.596), DDT (Spearman r_s_ = −0.20, n = 20, *p* = 0.417) and eggshell thickness of eggs analysed in 1977.In the eggs analysed in 2012, there was no significant correlation of eggshell thickness with DDE (Spearman r_s_ = 0.17, n = 20, *p* = 0.477) or with DDT (Spearman r_s_ = 0.09, n = 20, *p* = 0.696). There was also no significant correlation between DDE, DDT or sum14PCB and eggshell colour (R:G ratio) (DDT: Spearman r_s_ = 0.24, *p* = 0.311, DDE: r_s_ = −0.16, *p* = 0.504, PCB14: r_s_ = −0.30, *p* = 0.197). Current DDT concentrations in Faroese Great Skua eggs are now below the limit where we would expect to find an effect on eggshell thickness (Beyer and Meador 2011). The mean DDE concentration of 1823 ± 1213 µg/kg ww, is however within the range of concentrations where eggshell thinning would occur in some birds of prey (>100 µg/kg ww for Golden Eagles *Aquila chrysaetos*) (Newton and Galbraith 1991). However it is well established that bird species differ in their tolerance of DDE (Beyer and Meador 2011), and the marine diet is generally richer in calcium than in terrestrial ecosystem (Reynolds and Perrins 2010), so calcium may not become a limiting factor, despite the detrimental effects from DDE and DDT on calcium metabolism.

In addition to a decline in POP levels in Great Skua over a period of 35 years, our data also showed that Faroese eggs in 2012 had statistically higher POP levels than Shetland Great Skua eggs in 2008 (Table 1). There might be at least three reasons why Faroese Great Skuas have a higher pollutant load than Shetland Great Skuas: Difference in wintering areas with different pollutant loads, differences in atmospheric wet deposition of pollutants in their breeding area, and differences in diet. Recent studies have shown that wintering area can significantly influence the pollutant load of several seabird species in the breeding season (Bourgeon et al. 2013; Leat et al. 2013; Fort et al. 2014; Carravieri et al. 2014). However observations of ringed birds suggest that Faroese Great Skuas have similar wintering areas to the Great Skuas breeding in Shetland (Hammer et al. 2014). Between-population differences in POP levels may also be due to differences in atmospheric deposition of POPs in the birds’ breeding area. POPs vary in volatility which means that some compounds may travel far in gaseous form, and then precipitate in colder areas far from their source (Jones and de Voogt 1999). This is particularly exemplified by the accumulation of POPs in Arctic biota (de Wit et al. 2002). While it is not known whether the deposition rate of POPs differs between the Faroes and Shetland, the relatively close proximity and similarity in precipitation rates and ocean currents between these two sites could suggest there is another important factor to consider in relation to the difference of POPs concentrations between Faroes and Shetland Great Skuas.

All POPs accumulate through the food web, and organisms will potentially accumulate POPs to different extents, depending on their trophic position (Peakall 2007). Since the Great Skua occupies some of the highest trophic position in the Faroese marine ecosystem (feeding on fish, birds and mammals Furness 1987) it is therefore not surprising that it should accumulate high concentrations of pollutants as was found by Bloch et al. (1987) and in this study (Table 1). Studies of regurgitated pellets suggest that in Iceland, Shetland and further south, Great Skuas feed mainly on sandeels and fishery discards, while in the Faroes, Great Skuas feed mainly on other seabirds (Bayes et al. 1964; Leat 2012). Faroese Great Skua eggs had significantly higher δ^15^N values (12.54 ± 0.50) than Shetland eggs (11.77 ± 0.32) (Leat, unpublished): t = 6.77, *df* = 48, *p* < 0.001), suggesting Faroese skuas forage at a higher trophic level than Shetland skuas. Differences in stable isotope ratio in predators in different geographic regions may not only be due to differences in prey but due to geographic variation in baseline stable isotope values (Cherel and Hobson 2007). However, across Shetland, Iceland and Bear Island populations, differences in stable isotope values from blood samples were corroborated by between-population differences in diet inferred from analysis of pellet composition (Bourgeon et al. 2012).

We also investigated whether the trophic position measured in the eggs would correlate with POP levels within the Faroese population in 2012. As in Leat et al. (2011) we found no individual POP compound to correlate significantly with δ^15^N ratio in the eggs (Table 2). Furthermore, similar to Leat et al. (2011) all correlation coefficients were positive. Therefore within-population Great Skuas that feed on a higher trophic level might have higher levels of POPs in their eggs, although this is not statistically significant and between-population feeding on a higher trophic level had higher POP levels (Faroes) compared to the population feeding on a lower trophic level (Shetland). Possibly the differences in trophic level are larger between populations than within populations resulting in the difference in statistical significance between the two comparisons. Differences in diet between the Faroese and Shetland Great Skuas may also therefore help us to understand the marked difference in pollutant concentrations between Faroes and Shetland.

**Table 2:** Spearman rank correlations between POPs and δ^15^N ratio in homogenised Great Skua egg content

	Spearman’s Rho	*p* value
SumPCB	0.18	0.437
p,p′-DDE	0.05	0.831
p,p′-DDT	0.20	0.831
β-HCH	0.41	0.075
HCB	0.09	0.702*
Mirex	0.28	0.226
Cis-nonachlor	0.37	0.112
Trans-nonachlor	0.37	0.112
Oxychlordane	0.13	0.577
Toxaphene, Parlar no. 26	0.27	0.247
Toxaphene, Parlar no. 32	0.23	0.331
Toxaphene, Parlar no. 50	0.22	0.342*
Alpha-chlordane	0.29	0.214
PCB, Aroclor 1260	0.17	0.484

* Tied values

This study presents evidence that concentrations of some legacy POPs found in the Faroese environment have declined over the last 35 years. There were no detectable sub-lethal effects from these pollutants on the eggs of contemporary samples. However, the difference in POPs concentrations between the Faroes and Shetland lends support to the idea that the diet at the breeding area influences POPs exposure of Faroese Great Skuas more than other factors such as atmospheric deposition and wintering area. Whether the importance of geographically distinct diet specialisations for between-population variation in contaminant loads also holds for other systems remains to be explored.
